# Countenance and implication of Β-sitosterol, Β-amyrin and epiafzelechin in nickel exposed Rat: in-silico and in-vivo approach

**DOI:** 10.1038/s41598-023-48772-4

**Published:** 2023-12-04

**Authors:** Sara Zahid, Arif Malik, Suleyman Waqar, Fatima Zahid, Nusrat Tariq, Ali Imran Khawaja, Waqas Safir, Faisal Gulzar, Javeid Iqbal, Qurban Ali

**Affiliations:** 1 Institute of Molecular Biology and Biotechnology (IMBB), The University of Lahore, Lahore, Pakistan; 2 Ibadat International University (IIUI), Islamabad, Pakistan; 3 Xinjiang Key Laboratory of Biological Resources and Genetic Engineering, College of Life Sciences and Technology, Xinjiang University, Urumqi, 830046 Xinjiang China; 4 Faculty of Pharmacy, The University of Lahore, Lahore, Pakistan; 5 M. Islam Medical and Dental College, Gujranwala, Pakistan; 6 School of Pharmacy, Minhaj University Lahore, Lahore, Pakistan; 7 Department of Plant Breeding and Genetics, Faculty of Agricultural Sciences, University of the Punjab, Lahore, Pakistan

**Keywords:** Cancer, Cancer therapy

## Abstract

The detrimental impact of reactive oxygen species on D.N.A. repair processes is one of the contributing factors to colon cancer. The idea that oxidative stress may be a significant etiological element for carcinogenesis is currently receiving more and more support. The goal of the current study is to evaluate the anti-inflammatory and anticancer activity of three powerful phytocompounds—sitosterol, amyrin, and epiafzelechin—alone and in various therapeutic combinations against colon cancer to identify the critical mechanisms that mitigate nickel's carcinogenic effect. To evaluate the ligand–protein interaction of four selected components against Vascular endothelial growth factor (VEGF), Matrix metalloproteinase-9 (MMP9) inhibitor and Interleukin-10 (IL-10) molecular docking approach was applied using PyRx bioinformatics tool. For in vivo analysis, hundred albino rats were included, divided into ten groups, each containing ten rats of weight 160–200 g. All the groups were injected with 1 ml/kg nickel intraperitoneally per week for three months, excluding the negative control group. Three of the ten groups were treated with β-sitosterol (100 mg/kg b wt), β-amyrin (100 mg/kg b wt), and epiafzelechin (200 mg/kg b wt), respectively, for one month. The later four groups were fed with combinatorial treatments of the three phyto compounds for one month. The last group was administered with commercial drug Nalgin (500 mg/kg b wt). The biochemical parameters Creatinine, Protein carbonyl, 8-hydroxydeoxyguanosine (8-OHdG), VEGF, MMP-9 Inhibitor, and IL-10 were estimated using ELISA kits and Glutathione (G.S.H.), Superoxide dismutase (S.O.D.), Catalase (C.A.T.) and Nitric Oxide (NO) were analyzed manually. The correlation was analyzed through Pearson’s correlation matrix. All the parameters were significantly raised in the positive control group, indicating significant inflammation. At the same time, the levels of the foresaid biomarkers were decreased in the serum in all the other groups treated with the three phytocompounds in different dose patterns. However, the best recovery was observed in the group where the three active compounds were administered concomitantly. The correlation matrix indicated a significant positive correlation of IL-10 vs VEGF (r = 0.749**, *p* = 0.009), MMP-9 inhibitor vs SOD (r = 0.748**, *p* = 0.0 21). The study concluded that the three phytocompounds β-sitosterol, β-amyrin, and epiafzelechin are important anticancer agents which can target the cancerous biomarkers and might be used as a better therapeutic approach against colon cancer soon.

## Introduction

Numerous reports have provided evidence that the tumorous growth in the large intestine through the formation of polyps is known as colon cancer, the third most common type of Cancer in the United States^1^. Over half a million people die yearly from colon cancer^2^. The mortality rate is 8% of all cancers worldwide^3^. The data from 2017 provides evidence of 95,520 new diagnoses in the United States^4^. In Iran, the death rate by colon cancer is 6.3%, and in Tehran, the death rate is increased abruptly to 82% during the last 30 decades^5^. Not only the inherited factors but several environmental risk factors, such as a diet rich in processed foods, alcohol intake, and drinking water containing a high level of heavy metals such as nickel, also plays a potent role in the incidence of colon cancer^6^. Biochemical modulation of colon cancer by nickel is mediated through major control points in biochemical cascades such as D.N.A. methylation, binding of the receptor to the exogenous nickel, association of bound-nickel receptor complex with transcription factors, and activation of nickel-induced transcription factors leading to the oncogenic expression^7^. Human carcinogens include water-insoluble nickel sulfide (NiS), water-soluble nickel sulfate (NiSO4), and water-soluble nickel chloride (NiCl_2_)^8^. However, the latter two are more effective carcinogens than the former. Workers who work with nickel suffer very substantial D.N.A. damage^9^. Numerous human cell systems, including human hepatocellular carcinoma (HepG2)^10^. human TK6^11^, Chinese hamster lung fibroblast^12^, A375^13^ and HCT-116 cells^14^. Were also reported to be susceptible to D.N.A. damage caused by Ni^2+^ ions in earlier investigations^15^. Recent research on rat colonocytes has shown that cells underlying the lower crypt portion are more sensitive toward hydrogen peroxide damage than differentiated cells present at the surface of the crypt^16^. The intestinal mucosa is constantly exposed to diet, and heavy metal-derived oxidants and carcinogens result in redox imbalance and uncontrolled intestinal metabolic homeostasis with Cancer as an endpoint^17^. The serum of colon cancer patients shows increased levels of different markers of oxidative stress, such as increased levels of interleukins (I.L.s), matrix metalloproteinase (M.M.P.s)^18^, 8-hydroxydeoxyguanosine (8OHdG), HNE^19^, Glutathione peroxidase (GPx), superoxide dismutase (S.O.D.), catalase (C.A.T.)^20^. The biochemical modifications also increase renal factors, such as Creatinine, contributing to colon carcinogenesis^21^. The increased expressions of these enzymes also stimulate various signaling pathways associated with carcinogeneses, such as MAPK and NF-kB, resulting in increased expression of cellular defense-associated enzymes MnSOD, iNOS, eNOS, and GPx^22^.

Plant-based secondary metabolites have been the key to interest for scientists nowadays for treating various diseases, including Cancer, due to fewer side effects and improved efficacy compared to allopathic medicines and chemotherapy^23^. β-sitosterol is an active metabolite that is a saturated sterol in several medicinal plants such as *Parkinsonia acculenta*. It markedly inhibited the tumor progression and was observed to inhibit the lipase activity of D.N.A. polymerase β, resulting in the inhibition of D.N.A. repair synthesis^24^. *Gledista sinensis* was observed to isolate one terpenoid and four steroids, out of which β-sitosterol was the most active antitumor compound, showing 51.2% and 64.2% decrease in the carcinogenic parameters^25^. It was observed to ameliorate the levels of hepatic lipid peroxidation with an increase in the levels of antioxidants glutathione, superoxide dismutase, and catalase suggesting it is a powerful antioxidative and anticancer phytocompound^26^.

Similarly, β-amyrin is another active compound with antioxidative, antimicrobial, and anticancer properties. It is a triterpene of natural origin isolated from various sources, preferably from plant resins. Common sources of β-amyrin include *Byrsonima crassifolia, albizia lebbeck, resin of protium, and root bark of Ficus cordata.* It appeared responsible for the kidney's up-regulation of IFN-α, IL-4, and IL-17. NO and prostaglandins E2 were also suppressed in albino rat cases^27^. A recent study reported inhibiting prostate cancer cells' growth and showed anticancer effects against Hep-G2 liver cancer cells. This suggested β-amyrin, a potent anticancer compound. Over the years, flavonoids have attained considerable attention as bioactive molecules^28^. One flavonoid is epiafzelechin, found in green tea, grape, cocoa, *acacia chundra*, and many other plant species^29^. Epiafzelechin is reported to block angiogenesis, develop new capillaries, and suppress new cancer cell formation^30^.

In this association, the present work is aimed to evaluate the anti-inflammatory and anticancer activity of potent phytocompounds β-sitosterol, β-amyrin and epiafzelechin alone and in different therapeutic combinations against colon cancer to assess the key processes which are responsible for alleviating the cancerous effect of nickel.

## Materials and methods

### Data collection

Active constituents Beta-sitosterol, Beta-Amyrin, Epiafzelechin, and malondialdehyde were downloaded from the PubChem database in pdf format, and receptor VEGF (1FLT), MMP9 inhibitor (1GKC), and IL-10 (6X93) were downloaded from Protein Data bank in pdb format (www.pdb.org/pdb). The plant samples were purchased from the local market of Lahore. It has been confirmed that the experimental sample, including the collection of rats, complied with relevant institutional, national, and international guidelines and legislation with appropriate permission from the Ethical Review Committee of the Institute of Molecular Biology and Biotechnology (IMBB), The University of Lahore-Pakistan. It has been confirmed that the study was reported following ARRIVE guidelines.

### Protein preparation

Receptor proteins were opened in discovery studio software 2021(https://discover.3ds.com/discovery-studio-visualizer-download) to remove undesired substances. All the Heat atom, water molecules, and bounded ligands were removed from the protein; hydrogen atoms were added and were saved as in pdb format^31^.

### Ligand preparation

Ligands downloaded from the PubChem database (https://pubchem.ncbi.nlm.nih.gov/) are in pdf format, so they were uploaded in the Open Babel window of PyRx and were converted into pdb format^32^.

### Molecular docking

To evaluate the ligand–protein interaction of four selected components against VEGF, MMP9 inhibitor, and IL-10 molecular docking approach was applied using the PyRx bioinformatics tool (https://pyrx.sourceforge.io/)^33^. VEGF, MMP9 inhibitor, and IL-10 enzyme as receptor proteins were uploaded in PyRx individually and converted into a macromolecule. All ligand molecules were uploaded using the Open Babel window; the energy was minimized and converted into pdbqt format. Docking was run after creating a grad box using vina wizard (https://vina.scripps.edu/). The grad box dimension for VEGF X: 38.74, Y: 38.00, Z: 39.70, for IL-10, X: 98.67, Y: 132.83, Z: 136.47, and for MMP-INHIBITOR X: 57.72, Y: 52.72, Z: 80.73 was followed. Discover Studio Version 4.5 (https://www.discngine.com/discovery-studio) was used for results visualization including 2D structure, hydrogen bonding and bond length^34^.

### Drug-likeness and ADMET

SwissADMET online software (http://www.swissadme.ch/) was used for checking drug-likeness and ADMET analysis. ADMET is absorption, distribution, metabolism, and excretion^35^. Canonical SMILES of ligand compounds were retrieved from PubChem and were pasted in swissADMET to analyze drug-likeness and ADMET parameters.

### Toxicity

Canonical SMILES of all the ligands were retrieved from the PubChem database and were pasted in Protox-ii online web server (http://tox.charite.de/protox_II)^36^ and admetSAR (http://lmmd.ecust.edu.cn/admetsar2) for toxicity determination. Protox-ii online and admetSAR was run to check hepatotoxicity, mutagenicity and cytotoxicity, AMES toxicity, carcinogenicity, and acute oral toxicity of the compounds.

### Bioactivities

Orally administrated drugs need to be compatible with the drug-likeness properties to be finalized as pharmaceutically consistent with their bioactivity score, and that’s why we used Molinspiration Toolkit to predict selected compounds (https://www.molinspiration.com/cgi-bin/properties).

### In vivo analysis

Adult Wister male albino rats were taken from the Institute of Molecular Biology and Biotechnology, Lahore, Pakistan, with body weights ranging from 160 to 200 g. The Ethics Committee approved a study for Scientific Research at the University of Lahore (Approval No: U.S.M./Animal Ethics approval/2009/45/140). All animal experiments followed the Institutional Guidelines for the Care and Use of Animals for Scientific Purposes. A *p*-value of < 0.05 was considered statistically significant.

#### Plant extracts

The standardized 90% ethanolic bioactive extracts of Β-sitosterol and 80% ethanolic extracts of β-amyrin and epiafzelechin were purchased from the Sigma-Aldrich Corporation (St. Louis, MO, U.S.A.).

#### Experimental design

Hundred (100) albino rats were divided into ten groups. Every group had ten rats. The Group A rats were fed a normal diet and distilled water. For 12 weeks in a row, albino rats (*Rattus norvegicus*) of all groups excluding group A received an intraperitoneal injection of nickel chloride (1:1 v/v) along with saline (1 mL/kg b wt. per week) to cause colon damage, which was verified by biochemical tests. To assess the protective effects, Β-sitosterol (2.5 mg/ml/kg b wt), β-amyrin(2.5 mg/ml/kg b wt), and epiafzelechin (2.5 mg/ml/kg b wt)were fed orally to groups C, D, E, F, G, H and I for one month in different doses. Group J was treated orally with NALGIN (100 mg) 1tab/kg body weight that contains *Silybum marianum* (200 mg/ml/kg), *Picrorhiza kurroa* (50 mg/ml/kg), *Glycyrrhiza glabra* (50 mg/ml/kg) and *Cichorium intybus* (75 mg/mg/ml) (Table 1)^37^.

**Table 1:** Experimental Design.

GROUPS(n = 10)	Treatments
A	Control
B	Ni
C	Ni + β-Sitosterol
D	Ni + β-Amyrin
E	Ni + Epiafzelechin
F	Ni + β-Sitosterol + β-amyrin
G	Ni + β-Amyrin + Epiafzelechin
H	Ni + β-Sitosterol + Epiafzelechin
I	Ni + β-Sitosterol + β-Amyrin + Epiafzelechin
J	Ni + NALGIN©

Doses: Ni@ (1 mg/kg B.Wt. per week for three months).

β- Sitosterol,β-Amyrin, and Epiafzelechin @ (2.5 mg/Kg B.Wt./for six months).

NALGIN^**©**^ @ (500 mg/Kg B.Wt/day) for six months.

#### Tissue and serum separation

After the treatments, the animals were sacrificed by an intraperitoneal injection of ketamine (Arevipharma GmbH, Radebeul, Germany) (1 mL/g) as an anaesthesia. 5 mL blood was collected from the rat heart via cardiac puncture procedure into ethylene diamine tetra acetic acid (EDTA) treated sample bottles, and serum was separated by centrifugation at 1500 rpm for 10 min. After the serum separation, it was stored at – 60 °C until further biochemical analysis. The colon portions were separated and collected in falcon tubes containing 5% formalin for histopathological examination.

#### Biochemical assays

A commercially available Bio Merux and Randox kit was used to estimate Creatinine (C.R.T.) and Protein carbonyl (P.C.). Using commercially available ELISA kits, the levels of 8-hydroxy-2-deoxyguanosine (8-OHdG), matrix metalloproteinase-9 (MMP-9) inhibitor, vascular endothelial growth factor (VEGF) and Interleukin-10 (IL-10) were assessed (Abcam, Cambridge, MA, U.S.A.). Superoxide dismutase (S.O.D.) was estimated with the help of spectrophotometric method described by Kakkar et al.^38^ Glutathione (G.S.H.), catalase (C.A.T.), and nitric oxide (NO) were estimated by their respective spectrophotometric methods as explained by Moron et al.,^39^Sweazea et al.,^40^and Bredt and Synder^41^.

### Ethics approval

It has been confirmed that the experimental data collection complied with relevant institutional, national, and international guidelines and legislation with appropriate permissions from authorities of the Ethical Review Committee of the Institute of Molecular Biology and Biotechnology (IMBB), The University of Lahore-Pakistan.

## Results

### ADMET analysis

ADMET study was carried out using Swiss ADMET to identify different parameters like physical properties, lipophilicity, Lipinski, and solubility of bioactive constituents^42^. ADMET has a key role in drug development, as many drug candidates fail a clinical trial. No more than two violations are acceptable for oral drug candidates^43^. Lipinski rule of five (≤ 5 H- bond donor, ≤ 10 H-bond acceptors, Mol. weight ≤ 500 Da, Molar Refractivity 40–130 and ≤ 5logP)^44^ was followed for selected compounds to analyze whether these may act as drugs or not. The results in Table 1 show that all selected compounds pass the Lipinski rule of five, in which Epiafzelechin and Malondialdehyde have no single violation. In contrast, Beta-Amyrin and Beta-Sitosterol have one violation. Gastrointestinal absorption shows that two compounds, i.e., Epiafzelechin and Malondialdehyde, are highly soluble. For water solubility, Logs values range from − 10 to 0, indicating different solubility categories from − 10 to 0, i.e., − 10, − 6, − 4, − 2, and 0 for insoluble, poorly soluble, soluble, very soluble, and highly soluble, respectively^45^. Table 2 shows that all compounds are in the range of solubility, and no one is insoluble in the selected candidates.

**Table 2:** ADMET analysis and drug-likeness results of selected compounds.

Properties	Features	Beta-Aymrin	Beta-Sitosterol	Epiafzelechin
Physiochemical Properties	MW(g/mol)	426.72	414.71	274.27
	H.B. donor	1	1	4
	H.B. acceptor	1	1	5
	MR	134.88	133.23	72.31
	Arom. heavy Atom	0	0	12
	Rotatable bonds	0	6	1
	TPSA	20.23	20.23	90.15
Lipophilicity	logP(SILICOS-IT)	6.92	7.04	1.47
Water solubility	Logsw(SILICOS-IT)	− 7.16	− 6.19	− 2.72
Pharmacokinetics	G.I. absorption	Low	Low	High
Drug-likeness	Lipinski	Yes:1 violation	Yes:1 violation	Yes: 0 violation

### Toxicity

#### AdmetSAR

Online Protox-II server was used to analyze all constituents which may behave like a drug candidate to check their toxicity like hepatotoxicity, carcinogenicity, mutagenicity, and cytotoxicity. Today, toxicity prediction is a key step in drug designing to determine the unfavorable impacts of the compound on humans, plants, and the environment. Conventional approaches include different animal tests to assess the compound's toxicity, which is costly and time-consuming and includes serious ethical concerns. Computer-aided toxicity prediction is less time-consuming and inexpensive in comparison, provides results more frequently, and reduces the number of experimental biological tests. Table 3 shows that all selected candidates, i.e., Beta-Sitosterol, Beta-Amyrin, and Epiafzelechin, are non-toxic and may act as drug candidates. To validate and cross-check the toxicity of selected compounds, they were analyzed using admetSAR, which shows (Table 4) that three compounds with high docking scores are AMES non-toxic and non-carcinogenic.

**Table 3:** Toxicity results of selected compounds using Protox-II online server.

Compounds	Hepatotoxicity	Carcinogenicity	Mutagenicity	Cytotoxicity
Beta-Aymrin	Inactive	Inactive	Inactive	Inactive
Beta-Sitosterol	Inactive	Inactive	Inactive	Inactive
Epiafzelechin	Inactive	Inactive	Inactive	Inactive

**Table 4:** Toxicity profiling of selected compounds (with high binding affinity) using admetSAR server.

Compounds	hERG inhibition	Rat(LD50,mol/kg)	AMES	Carcinogen	Acute oral toxicity	Carcinogenicity (class 3)
Beta-Aymrin	Weak	2.0842	Non	Non	III	Non-required
Beta-Sitosterol	Weak	2.6561	Non	Non	I	Non-required
Epiafzelechin	Weak	2.0532	Non	Non	IV	Non-required

Further, all compounds were weak inhibitors for the human ether-a-go-go-related gene (hERG) and showed weak rat acute toxicity with a median lethal dose (LD50) of 2.26 mol/kg. As per the predicted acute oral toxicity, Beta-Amyrin lies in 'class III.' Compounds of this class have LD50 values < 5000 mg/kg and were generally considered suitable from a druggable point of view.

### Bioactivities

One of the greatest tools for assessing the bioactivity scores of natural chemicals for therapeutic targets is Molinspiration, as demonstrated in (Table 5). The likelihood that a compound has notable biological activity depends on its bioactivity score. Compounds with bioactivity values above 0.00 are most likely noteworthy, while those with scores between -0.50 and 0.00 are moderately dynamic, and those below − 0.50 are inactive. Our analysis demonstrates that many routes may be implicated in the chemical compounds' physiological actions. Interactions with nuclear receptor ligands, GPCR ligands, ion channel modulator ligands, enzyme inhibitors, and protease inhibitors might also bring it on. The bioactivity scores (Table 5) show that Beta-Amyrin, Beta-Sitosterol, and Epiafzelechin are highly active ligands of Nuclear Receptor and Beta-Amyrin Epiafzelechin act as highly active ligands of Enzyme inhibitors. Furthermore, the results show that Beta-Amyrin, Beta-Sitosterol, and Epiafzelechin are moderately dynamic toward GPCR ligand and Protease inhibitors. Beta-Sitosterol and Epiafzelechin are moderately dynamic toward Ion Channel, while Enzyme inhibitor Epiafzelechin shows moderate dynamics.

**Table 5:** Bioactivity score of selected compounds according to Molinspiration cheminformatics software.

Bioactivities	Beta-Aymrin	Beta-Sitosterol	Epiafzelechin
GPCR ligand	**0.22**	**0.14**	**0.38**
Ion channel modulator	− 0.05	**0.04**	**0.15**
Kinase inhibitor	− 0.31	− 0.51	**0.05**
Nuclear receptor ligand	*0.67*	*0.73*	*0.57*
Protease inhibitor	**0.11**	**0.07**	**0.25**
Enzyme inhibitor	*0.56*	**0.51**	*0.47*

Bioactivity score > 0 = active.

Bioactivity score − 0.5–0.0 = moderately active.

Bioactivity score < − 0.5 = Inactive.

Significant values are in bold and italic.

### Docking

**Figure 1:**
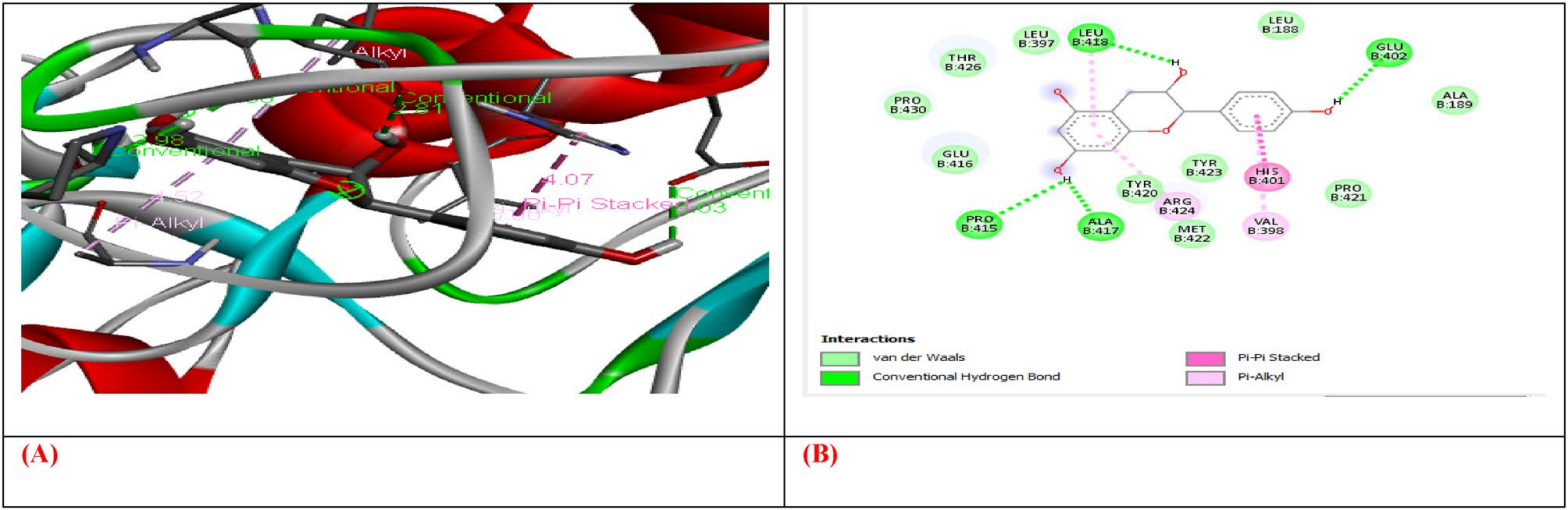
(**A**) shows the interaction of Epiafzelechin with receptor protein MMP9-inhibitor, while (**B**) shows the 2D interaction and bonding. Discover Studio version 4.5 was used to develop the structure (https://www.discngine.com/discovery-studio).

**Figure 2:**
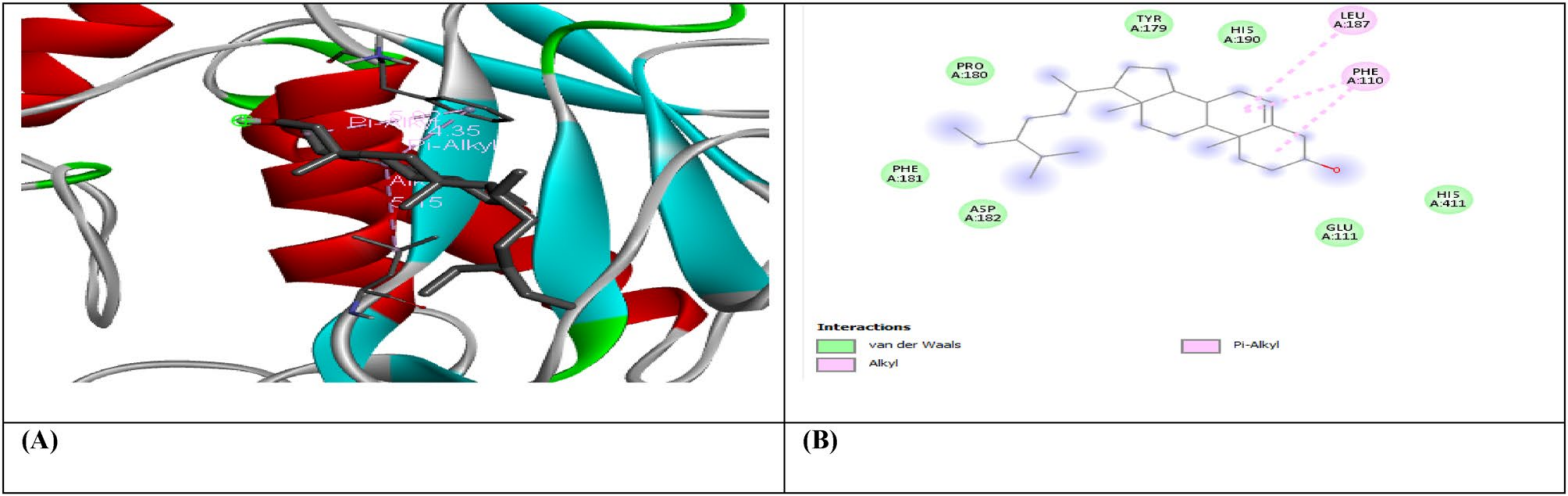
(**A**) shows the interaction of Beta-Sitosterol with receptor protein MMP9-inhibitor, while (**B**) shows the 2D interaction and bonding. Discover Studio version 4.5 was used to develop the structure (https://www.discngine.com/discovery-studio).

**Figure 3:**
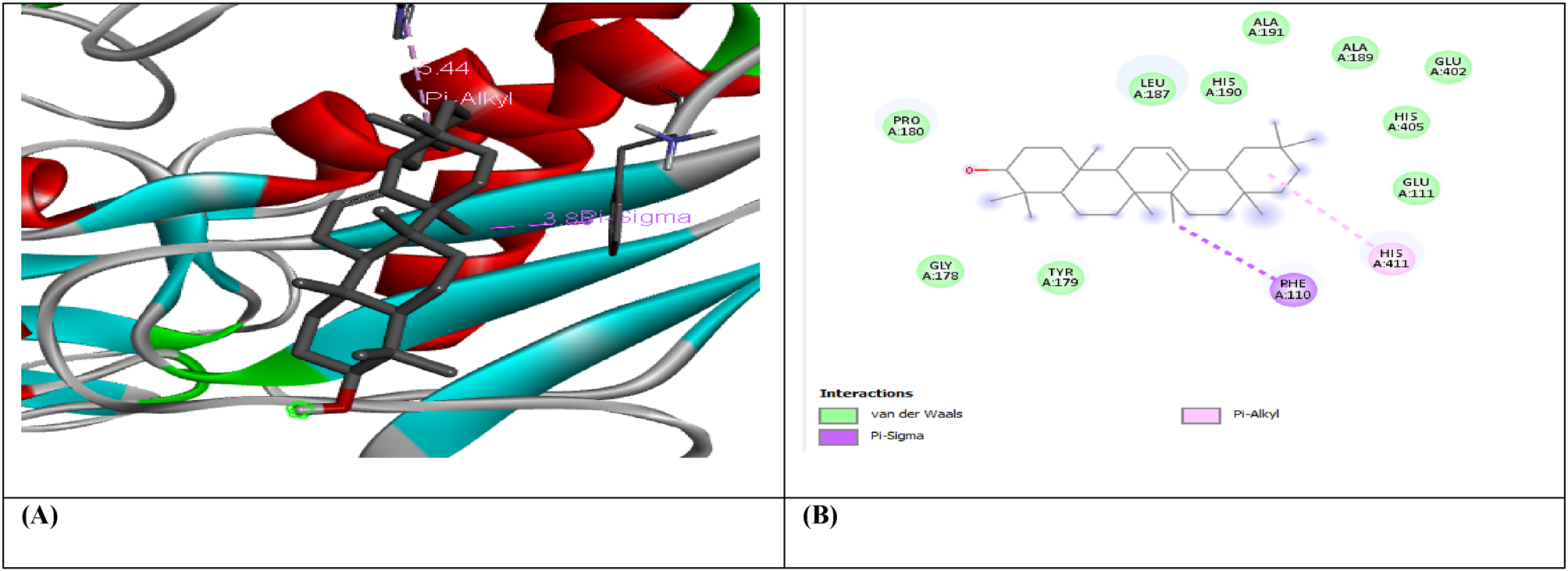
(**A**) shows the interaction of Beta-Amyrin with receptor protein MMP9-inhibitor, while (**B**) shows the 2D interaction and bonding. Discover Studio version 4.5 was used to develop the structure (https://www.discngine.com/discovery-studio).

**Figure 4:**
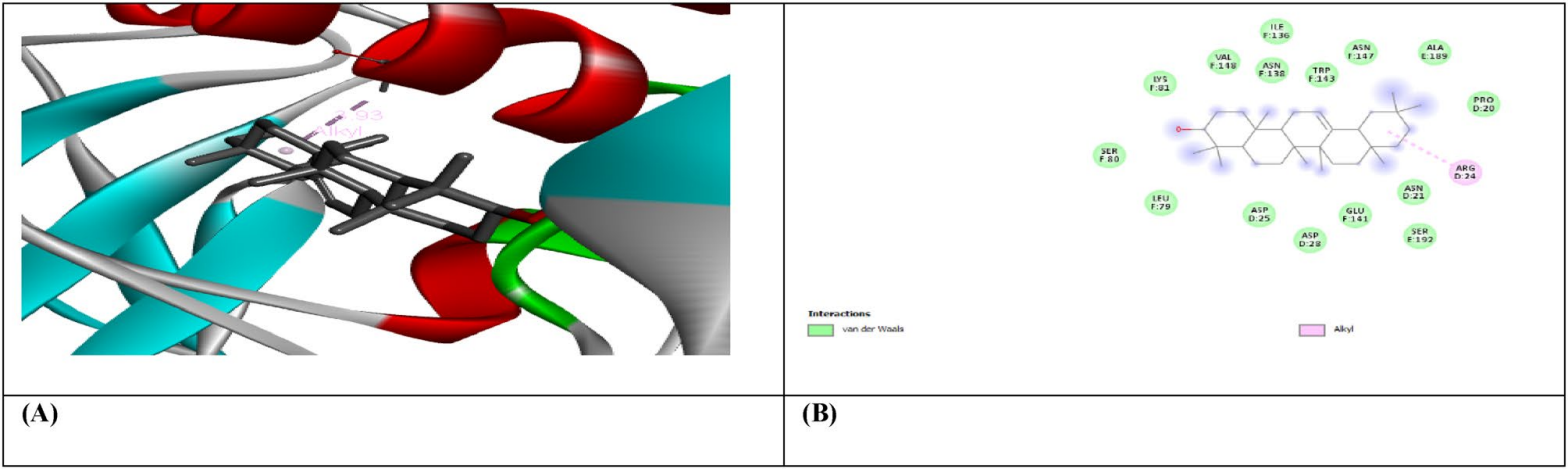
(**A**) shows the interaction of Beta-Amyrin with receptor protein IL-10, while (**B**) shows the 2D interaction and bonding. Discover Studio version 4.5 was used to develop the structure (https://www.discngine.com/discovery-studio).

**Figure 5:**
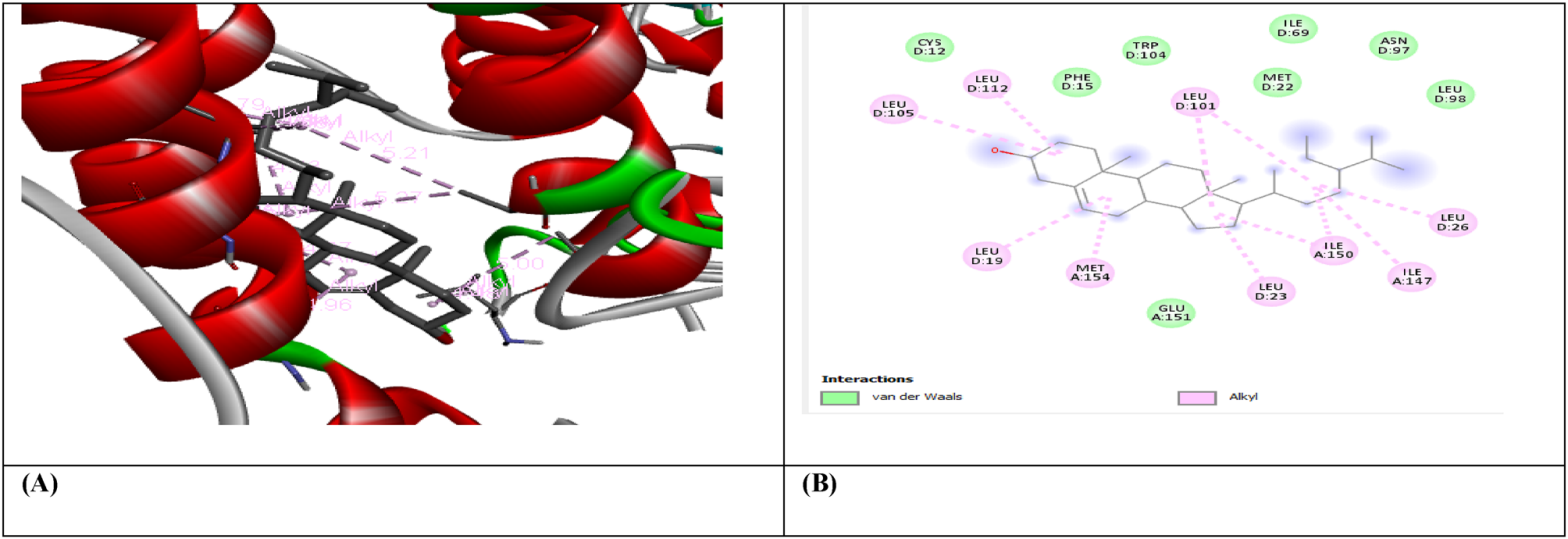
(**A**) shows the interaction of Beta-Sitosterol with receptor protein IL-10, while (**B**) shows the 2D interaction and bonding. Discover Studio version 4.5 was used to develop the structure (https://www.discngine.com/discovery-studio).

**Figure 6:**
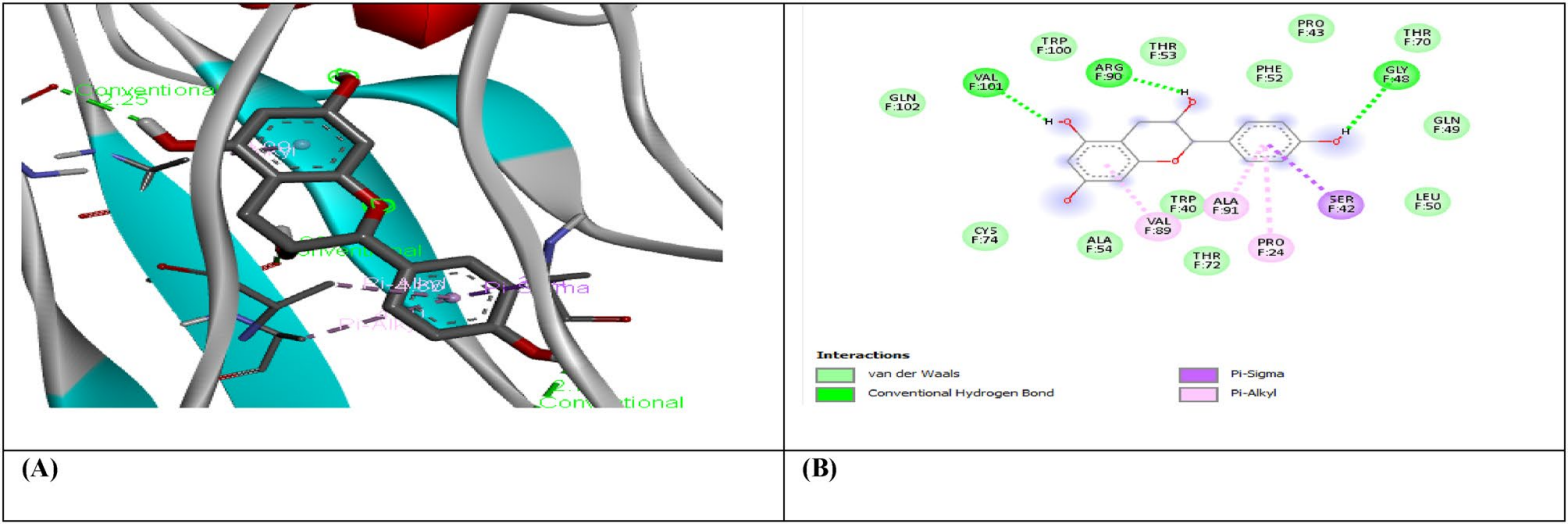
(**A**) shows the interaction of Epiafzelechin with receptor protein IL-10, while (**B**) shows the 2D interaction and bonding. Discover Studio version 4.5 was used to develop the structure (https://www.discngine.com/discovery-studio).

**Figure 7:**
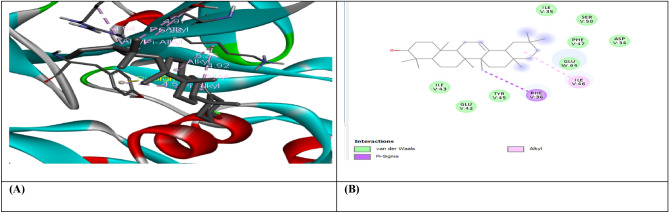
(**A**) shows the interaction of Beta-Amyrin with receptor protein VEGF, while (**B**) shows the 2D interaction and bonding. Discover Studio version 4.5 was used to develop the structure (https://www.discngine.com/discovery-studio).

**Figure 8:**
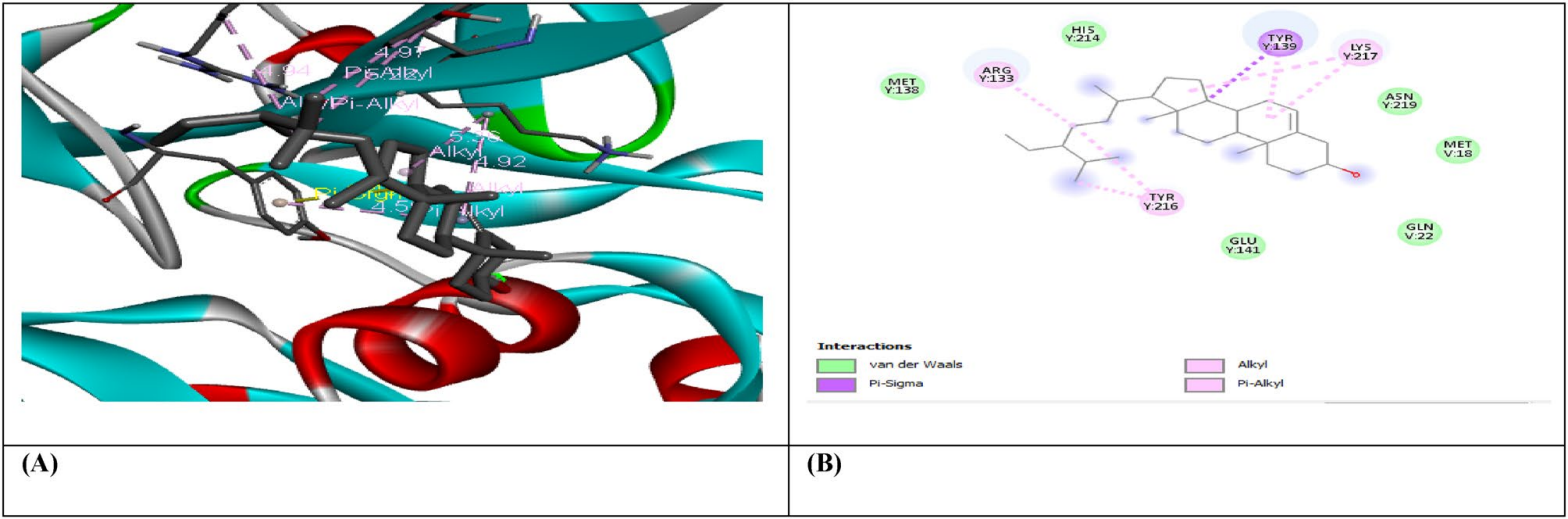
(**A**) shows the interaction of Beta-Sitosterol with receptor protein VEGF, while (**B**) shows the 2D interaction and bonding. Discover Studio version 4.5 was used to develop the structure (https://www.discngine.com/discovery-studio).

**Figure 9:**
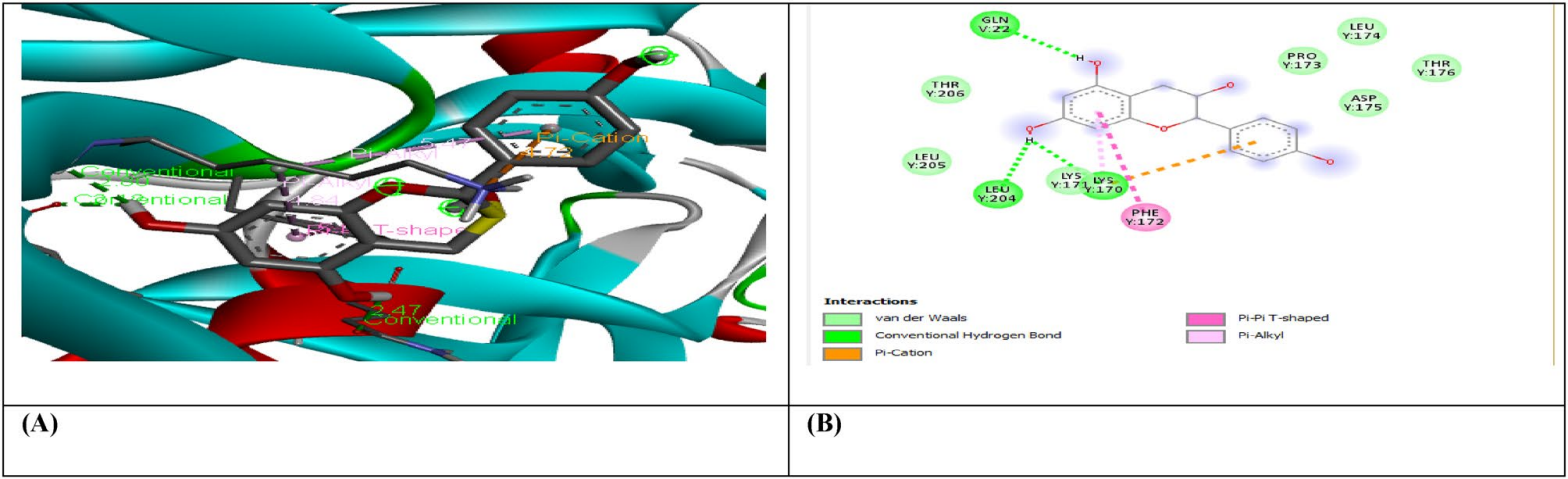
(**A**) shows the interaction of Epiafzelechin with receptor protein VEGF, while (**B**) shows the 2D interaction and bonding. Discover Studio version 4.5 was used to develop the structure (https://www.discngine.com/discovery-studio).

Molecular docking was carried out for selected compounds against VEGF, IL-10, and MMP9-Inhibitor to evaluate their therapeutic potential as anticancer agents (Figs. 1, 2, 3, 4, 5, 6, 7, 8, 9). Molecular docking has a key role in ligand–protein and protein–protein interaction to see for best pose and binding sites of receptors to which a ligand molecule binds and is extensively used in modern drug discovery^6^. Table 6 shows that three out of four selected compounds have a high binding affinity and low binding energy, which shows their strong interaction with receptor proteins VEGF, IL-10, and MMP9-Inhibitor. The results show that B-Amyrin and Beta-Sitosterol have a high binding affinity of − 8.7 and − 7.2 kcal/mol with VEGF, respectively. It is evident from the study that ARG133, TYR126 (2), LYS217 (2), TYR139 (2), PHE36, and ILE46 residues of VEGF enzyme interact with our selected compounds of Beta-Amyrin and Beta-Sitosterol. B-Amyrin, Beta-Sitosterol, and Epiafzelechin have a binding affinity of − 9.1 kcal/mol,  8.2 kcal/mol, and − 8.0 kcal/mol with IL-10, respectively. The results show that amino acid residues ARG24, MET154, LEU19, LEU105, LEU112, LEU101, LEU26, ILE147, ILE150, LEU23, VAL101, ARG90, GLY48, PRO24, ALA91, VAL89 and SER42 of IL-10 is interacting with our selected compounds. β-Amyrin and Epiafzelechin have a binding affinity of − 8.0 kcal/mol and − 9.2 kcal/mol with MMP9-Inhibitor, respectively. The results show that amino acid residues ALA147, PRO145, LEU418 (2), GLU402, ARG424, VAL398, HIS401, HIS411 and PHE110 of MMP9-Inhibitor is interacting with our selected compounds.

**Table 6:** Molecular docking results of selected compounds.

S.NO	Compounds	Receptors	Binding affinity (Kcal/mol)	Bonding type	Bond category	Active amino acid residues
1	Beta-Amyrin	VEGF	− 8.7	Hydrophobic	Pi-Sigma	PHE V:36
					Alkyl	ILE V:46
2	Epiafzelechin		− 6.6	Hydrogen	Conventional H.B	GLN V:22, LEU Y:204, LYS Y:170
				Hydrophobic	Pi-Alkyl	LYS Y:170
					Pi-Cation	LYS Y:170
					Pi-Pi-T shaped	PHE Y:172
3	B-Sitosterol		− 7.2	Hydrophobic	Pi-Alkyl	ARG Y:133, TYR Y:126, TYR Y:126, TYR Y:139
					Alkyl	LYS Y:217(2)
					Pi-Sigma	TYR Y:139
1	Beta-Amyrin	IL-10	− 9.1	Hydrophobic	Alkyl	ARG D:24
2	Epiafzelechin		− 8.2	Hydrogen	Conventional H.B	VAL F:101, ARG F:90, GLY F:48
				Hydrophobic	Pi-Alkyl	PRO F:24, ALA F:91, VAL F:89
					Pi-Sigma	SER F:42
3	B-Sitosterol		− 8.0	Hydrophobic	Alkyl	MET A:154, LEU D:19, LUE D:105, LEU D:112, LEU D:101 (2), LEU D:26, ILE A:147, ILE A:150, LEU D:23
1	Beta-Amyrin	MMP9-INHIBITOR	− 8.0	Hydrophobic	Pi-Alkyl	HIS A:411
					Pi-Sigma	PHE A:110
2	Epiafzelechin		− 9.2	Hydrogen	Conventional H.B	ALA B:147, PRO B:145, LEU B:418, GLU B:402
				Hydrophobic	Pi-Alkyl	LEU B:418, ARG B:424, VAL B:398
					Pi-Pi Stacked	HIS B:401
3	B-Sitosterol		− 6.6	Hydrophobic	Pi-Alkyl	PHE A:110
					Alkyl	LEU A:187

### In vivo biochemical response of *β-sitosterol*, *β-amyrin* and *epiafzelechin* in nickel-exposed rat

Depending on the colon microenvironment, several cytokines are responsible for the modulation of cellular transformation, their relative concentrations in the serum, cytokine receptor expression levels, and the surrounding cells' commencement status during chronic inflammation. Table 7 depicts the tumoral response of Creatinine, Protein carbonyl, and 8-hydroxydeoxyguanosine (8OHdG) during the progression of colon carcinogenesis in the intestine. The renal parameters Protein carbonyl and Creatinine were 39% and 12.2%, respectively, higher in rats receiving nickel (group B) than the normal group of rats (group A), indicating the malignant potential of the colon. Similarly, other inflammatory parameters’ levels of 8OHdG (3.1%) were also significantly higher in nickel-intoxicated rats than in the negative control group (group A). However, the sole treatment of the standardized active constituents β-sitosterol, β-amyrin, and epiafzelechin showed antitumor effects with 77%, 93%, and 90% recovery compared to the positive control group (group B). Different combinations of these active phytocompounds show significant (*p* ≥ 0.05) restoration with 57.9% in 8OHdG. The increased production of 8OHdG induces a tumour phenotype based on generating reactive oxygen species and reactive nitrogen species, which induce DNA damage, facilitating tumorigeneses. Raised serum 8OHdG is a hallmark of DNA. damage because 8oxodG can pair with adenine, leading to the transversion of G: C to T: A (GT transversion). The best restoration was obtained through the combinatorial regimen of three phytocompounds (group I) with 72% 8OHdG, 64% creatinine, and 67% A.L.T. compared to the commercially used anti-inflammatory drug Nalgin containing *silymarin, Glycyrrhizin, and Cichorium minibus.* Table 8 portrays the picture of the antioxidative status of superoxide dismutase (S.O.D.), glutathione (G.S.H.), and catalase (C.A.T.) and the carcinogenic status of Matrix metalloproteinase (MMP-9 inhibitor) in rats intoxicated with nickel and groups treated with different combinations of the three phytocompounds β-sitosterol, β-amyrin, and epiafzelechin. S.O.D., G.S.H., and C.A.T. were lower in rats administered with nickel (52%, 30.9%, and 17.25%, respectively) than in the negative control group. However, the levels of MMP-9 inhibitor were raised by 45% in the serum compared to the normal control group. The sole treatments of three bioactive compounds triggered significant (*p* ≥ 0.05) improvement in the antioxidative status (CAT. 67%, SOD. 56%, and GSH. 45%) and MMP—9 inhibitor, 78%, as compared to the positive control group (Group B). Matrix metalloproteinases (MMPs) comprise a multigene family of zinc-dependent extracellular matrix (ECM) remodelling endopeptidases implicated in pathological processes, such as carcinogenesis. In this regard, their activity is pivotal in tumour growth and the multistep processes of invasion and metastasis, including proteolytic degradation of ECM, alteration of the cell–cell and cell-ECM interactions, migration and migration, and angiogenesis. The best recovery was obtained in the group of rats treated with the combinatorial regimen of the three active antitumor compounds (group I), with an increase of 78% in SOD, 89% in GSH, 93% in CAT and a decrease of 58% in NO and 83% in MMP-9 inhibitor protein as compared to the commercially used standard drug NALGIN.

**Table 7:** Biochemical response of β-sitosterol, β-amyrin, and epiafzelechin in rat model following nickel-induced toxicity.

Groups	PC	Creatinine	8-OHdG	VEGF	IL-10
	µmol/ml	mg/L	pg/ml	pg/ml	pg/ml
A	3.99 ± 1.23	0.61 ± 0.013	0.98 ± 0.089	61.25 ± 8.26	18.26 ± 4.26
B	10.23 ± 2.07	4.99 ± 1.22	31.32 ± 6.16	102.26 ± 12.26	41.26 ± 4.65
C	8.26 ± 3.45	3.26 ± 1.10	27.16 ± 3.26	96.35 ± 6.35	39.65 ± 1.99
D	7.26 ± 2.08	4.01 ± 1.16	31.25 ± 6.25	91.35 ± 5.23	36.35 ± 2.18
E	8.56 ± 1.88	3.11 ± 0.84	29.36 ± 4.16	85.26 ± 8.16	31.28 ± 3.88
F	6.55 ± 2.08	2.16 ± 0.99	21.25 ± 2.16	71.23 ± 6.35	29.35 ± 2.65
G	5.66 ± 1.04	2.01 ± 0.65	18.16 ± 3.26	68.35 ± 4.25	23.25 ± 1.88
H	4.99 ± 1.11	1.98 ± 0.19	17.26 ± 1.99	66.35 ± 1.99	21.26 ± 3.18
I	4.08 ± 2.14	0.99 ± 0.08	5.16 ± 1.28	64.35 ± 3.28	20.65 ± 1.47
J	5.99 ± 2.33	1.26 ± 0.25	9.26 ± 2.88	71.26 ± 5.66	25.26 ± 2.18
LSD (0.05)	1.25	1.08	4.16	10.26	1.29
*p*-VALUE	0.029	0.031	0.013	0.014	0.033

**Table 8:** Biochemical response of β-sitosterol, β-amyrin, and epiafzelechin in rat model following nickel-induced toxicity.

Groups	SOD	GSH	CAT	NO	MMP-9 inhibitor
	IU/ml	µmol/ml	µmol/ml of Prot	µmol/L	pg/ml
A	1.19 ± 0.62	8.26 ± 2.26	6.26 ± 1.55	19.26 ± 1.99	44.26 ± 8.26
B	0.62 ± 0.012	2.56 ± 0.56	1.08 ± 0.012	41.26 ± 5.26	96.35 ± 8.16
C	0.95 ± 0.045	2.99 ± 1.08	1.25 ± 0.56	36.25 ± 4.26	80.26 ± 6.35
D	0.85 ± 0.019	3.26 ± 1.03	1.65 ± 0.95	31.09 ± 3.33	74.26 ± 5.45
E	0.73 ± 0.021	4.16 ± 2.08	2.09 ± 0.89	28.26 ± 4.16	61.35 ± 4.44
F	0.99 ± 0.053	5.16 ± 1.01	2.08 ± 0.18	26.35 ± 3.26	56.35 ± 2.08
G	0.89 ± 0.088	5.08 ± 1.33	3.06 ± 1.07	23.33 ± 1.88	51.26 ± 4.47
H	1.001 ± 0.032	6.25 ± 2.09	3.96 ± 1.22	21.56 ± 2.99	49.65 ± 5.66
I	1.09 ± 0.051	7.88 ± 1.09	4.89 ± 2.07	20.59 ± 3.19	48.56 ± 6.35
J	0.812 ± 0.031	6.35 ± 1.22	2.88 ± 1.11	31.55 ± 1.88	55.26 ± 4.25
LSD (0.05)	0.512	1.02	0.956	5.26	4.16
*p*-VALUE	0.019	0.017	0.027	0.031	0.00

**Figure 10:**
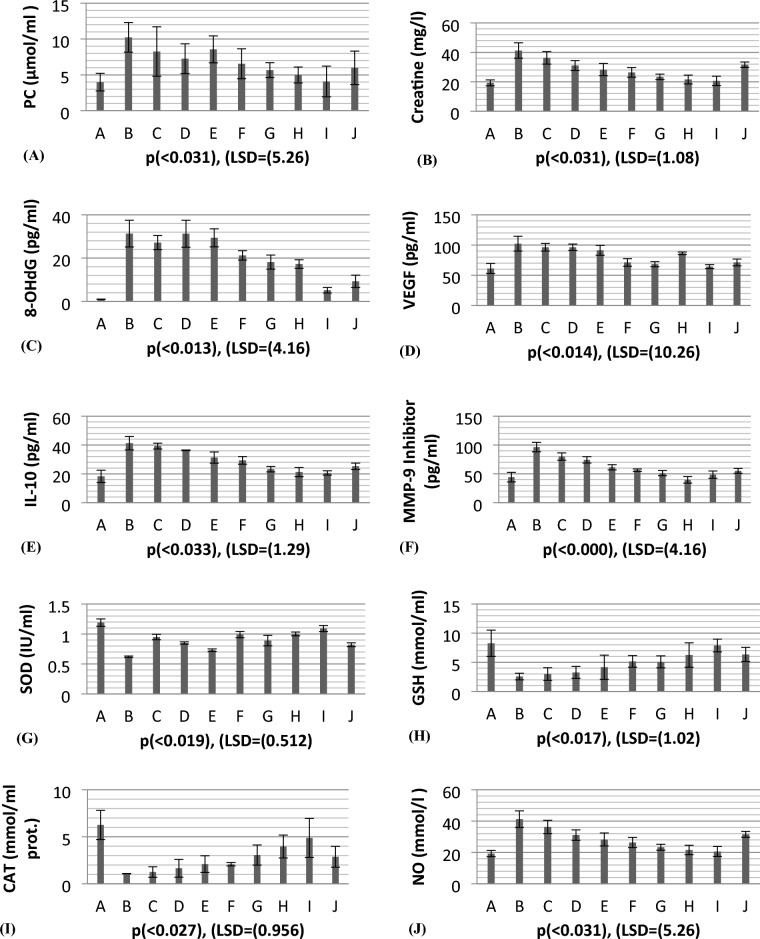
Cellular response of *β*-Sitosterol, *β*-Amyrin, and epiafzelechin in the nickel-induced rat model.

Table 7 shows the variation of crucial colon cancer vascular endothelial growth factor (VEGF) and interleukins (IL- 10) biomarkers. There is significant (*p* ≥ 0.05) elevation found in the levels of VEGF (59%) and IL-10 (44.2%) in the group of rats intoxicated with nickel as compared to the normal healthy rats. Higher levels of IL-10 are associated with cellular stress and macromolecule modification, thus regulating the signaling pathways of cellular proliferation through Akt, Erk1/2, and hypoxia-inducible factor-1 (HIF-1) activation. In this concept, the best restoration in the levels of foresaid biomarkers (VEGF) and IL-10 (76.8%) was obtained by the co-treatment of β-sitosterol, β-amyrin, and epiafzelechin as compared to NALGIN-treated rats (Fig. 10).

IL-10 is also an angiogenic factor highly correlated with VEGF in colon cancer (r = 0.749, *p* = 0.009), as IL-10 induces VEGF expression dose-dependently. This means that the increase in VEGF value will also cause an increase in IL-10 in a dose-dependent manner. Similarly, a significant correlation was also depicted in Table 9 between IL-10 and 8-OHdG (r = 0.648, *p* = 0.035), VEGF, and 8-OHdG (r = 0.648, *p* = 0.032). Both are the hallmarks of cellular stress and inflammation (Table 9). The direct relationship between MMP-9 and CAT. (r = 0.679,*p* = 0.041) and with SOD. (0.748, *p* = 0.021) was observed, which means that when colon inflammation increased, the increased levels of MMP-9 caused the elevation of SOD. and CAT. levels in the serum.

**Table 9:** Pearson s’ correlation coefficients of different variables in colon tissues of rats under nickel stress receivingβ-sitosterol, β-amyrin, and epiafzelechin.

VARIABLES	R	*P* (< 0.05)
IL-10 Vs. VEGF	0.749	0.009
VEGF Vs. 8OHdG	0.648	0.032
IL-10 Vs. 8OHdG	0.648	0.035
MMP-9 Vs. CAT	0.679	0.041
MMP-9Vs. SOD	0.748	0.021

### Histopathological analysis

**Figure 11:**
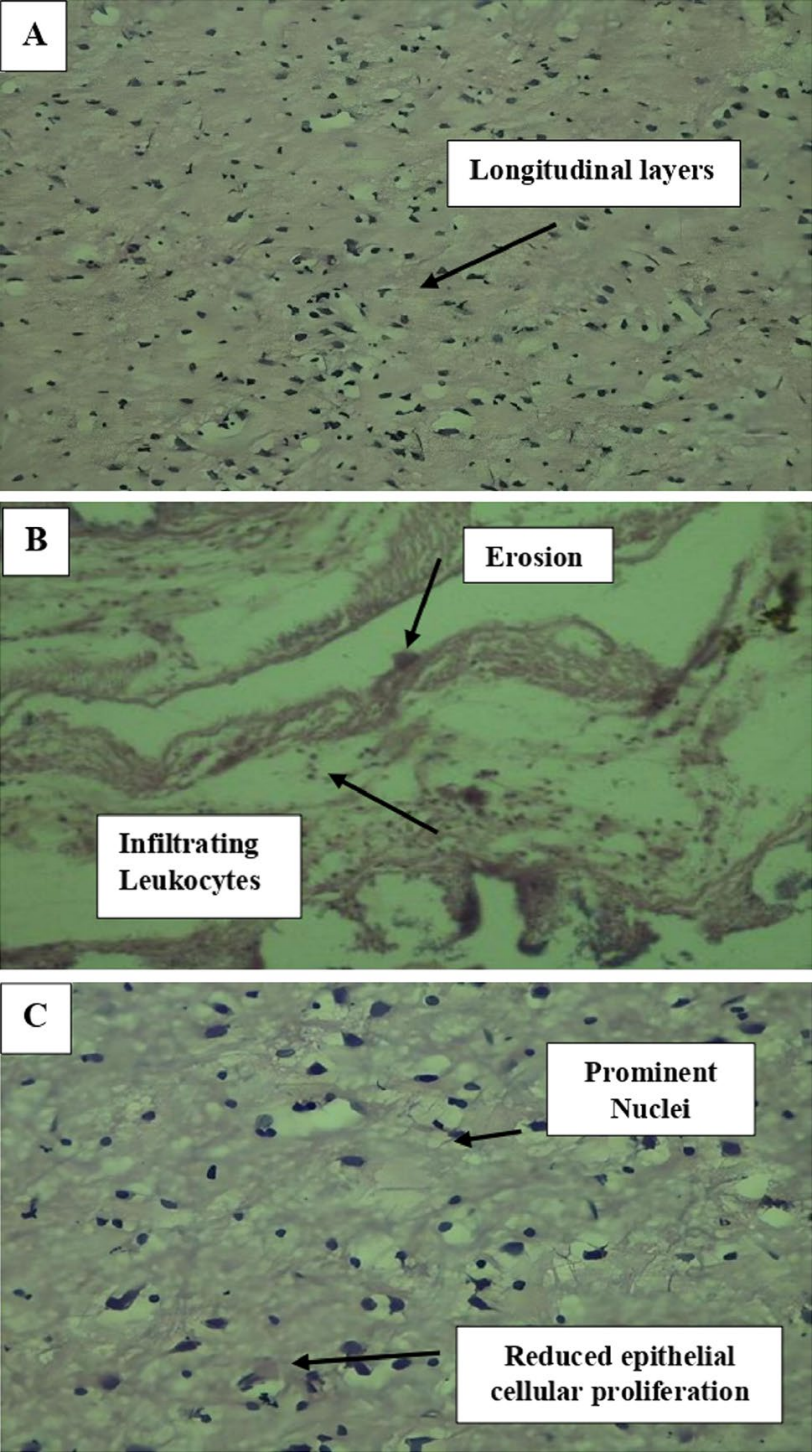
Regular connective tissue with well-demarcated blood vessels and numerous goblet cells. The muscular muscosae are a bit more prominent and consist of distinct inner circular and outer longitudinal layers (**A**). Erosions, i.e. the loss of surface epithelium with underlying inflammation where the epithelial defect reaches the basement membrane. Infiltrating leukocytes i.e. granucloytes, monocytes and lymphocytes, were common in nickel-affected inflamed intestines (**B**). A muscular externa containing inner circular and outer longitudinal smooth muscle layers. Note the inflamed coli are not present in this section of colon. Lymphatic nodules in the lamina propria and submucosa can be seen clearly. No epithelial cell proliferation was observed (**C**).

Evaluation of disease activity and severity, as well as reporting treatment outcomes in clinical trials, are essential for the clinical management of colon cancer. The treatment's most obvious goal is to control inflammation and achieve complete symptom remission. The histopathological analysis depicted the typical normal colonic mucosa composed of many goblet cells that release mucin. There is a submucosa underneath. Tiny lymphoid nodules linked with the intestines could also be observed (Fig. 11A). The presence of neutrophils indicates the existence of inflammatory activity. Active inflammation is characterized by neutrophilic cryptitis, crypt abscesses, bleeding, erosions, ulceration, and necrosis. The architectural distortion is more pronounced, characterized by the irregular spacing of the crypts resulting from the inflammatory cells' lamina propria expansion and basal lymphoid aggregates (Fig. 11B). However, inflamed coli are reduced in the right side figure with 30 to 40% restoration of rectal mucosa can be observed due to the treatment with a combinatorial dose of β- sitosterol, β-amyrin, and epiafzelechin (Fig. 11C). The epithelial cellular proliferation was also reduced due to the combinatorial treatment. These histopathological results also agree with our in vivo results (Table 7). This indicated the best colon necrosis and ulceration recovery due to the synergistic dosage regimen of the foresaid phytocompounds.

## Discussion

Nickel contamination in large quantities of food or water can be extremely hazardous^46^. Cancer initiation and development are frequently linked to nickel-induced apoptosis inhibition in colon cells^47^. Consequently, a thorough understanding of apoptotic signalling pathways is related to discovering target-selective therapeutic medicines. The current study aimed to assess how the phytoactive compounds *β-sitosterol*, *β-amyrin*, and *epiafzelechin* in different dosage combinations combat the nickel-affected adult Wistar rats' ascending colons. The two main apoptotic events are morphological and metabolic changes in the cancerous colon cells^48^. Cell morphological changes, including cell shrinkage, chromatin condensation, membrane blebbing, and nuclear disintegration, which later result in the formation of apoptotic bodies, arise after triggering biochemical signals in cell^49^. In this study, analysis of the Creatinine, protein carbonyl, 8-OHdG, VEGF, S.O.D., G.S.H., C.A.T., NO, MMP-9 Inhibitor, and IL-10 was carried out to investigate the nickel-induced biochemical changes in colon cancer cells. The intraperitoneal induction of NiCl_2_ in rats caused significant elevation to the serum levels of Creatinine and P.C. in a manner reminiscent of compromised colon integrity. However, the intraperitoneal administration of different concentrations of *β-sitosterol*, *β-amyrin*, and *epiafzelechin* in rats prevented the elevation of the indices for colon dysfunction. These findings support previous studies showing that *β-sitosterol*, *β-amyrin*, and *epiafzelechin* offered colon and hepatoprotection^50,51^. Also, in a recent study, the protective effect of *β-sitosterol*, *β-amyrin*, and *epiafzelechin* against lead-induced colorectal toxicity has been demonstrated^52^. The evidence supports the notion that including the above-mentioned phytoactive compounds in diets may enhance the health of the inflamed or cancerous colon.

8-hydroxydeoxyguanosine (8OHdG) has been the hallmark of cancer^53^ and oxidative D.N.A. damage^54^. The nickel administered in the rats in our study caused D.N.A. methylation, which is a post-translational modification during the synthesis of cancerous colon protein and D.N.A. adducts 8OHdG and 8-oxodG, both are also the major products of D.N.A. oxidation. Nalgin tablets used as a standard commercial drug in the study couldn't combat the inflammation caused by D.N.A. damage due to the formation of D.N.A. adducts 8OHdG and 8-oxodG (Table 7). The increased levels of 8OHdG (31.32 pg/ml) as compared to normal 0.98 pg/ml in rats (Table 7) is the main risk factor for developing colon cancer which is subsequently decreased by the co-administration of the foresaid bioactive compounds (5.16 pg/ml). This is supported by Pearson’s correlation (Table 9) between COX-2 and 8-OHdG (r = 0.648**, *p* = 0.035). Similarly, VEGF, IL-10, and MMP-9 inhibitor proteins are all increased due to free radical generation in the pathogenesis of colon cancer^55^. The combination regimen of the three phytocompounds in group I lowers the levels of these biomarkers of D.N.A. oxidation compared to the isolated treatment. The correlation matrix shows the significant and direct relationship of IL-10 and VEGF (r = 0.749**, *p* = 0.009), MMP-9, and C.A.T. (r = 0.679*, *p* = 0.041). These results are also in accordance with the in silico analysis of VEGF with the three bioactive compounds showing strong binding affinity of − 8.7 with β-amyrin, − 6.6 with epiafzelechin, and − 7.2 with β-sitosterol (Table 6) which means that these bioactive compounds used as drugs in the current study bind strongly in the active pockets of VEGF protein, consequently controlling its expression to control the disease. This suggests that these phytoactive compounds are more potent drugs than others used in chemotherapy. The significant correlation between the foresaid parameters depicts that all these parameters are the prominent biomarkers of colon tumorigeneses which were significantly restored by the synergistic treatment of the three bioactive compounds. The matrix metalloproteinases are the most potent proteinases associated with tissue homeostasis in normal cell circumstances^56^. However, the over-expression of the MMP-9 inhibitor protein causes a change in proteolysis due to angiogenesis resulting in metastatic colon tumor invasion and inflammation^57^. In our study, we also analyzed the data statistically through Pearson's correlation matrix to know the relationship of the said variables, which helped us to identify and visualize the large dataset pattern of the severity of the disease. The correlation matrix shows the significant and indirect relationship between MMP-9 inhibitor protein and S.O.D. (r = 0.748**, *p* = 0.021). In our study, the modulation of MMP-9 inhibitor protein is inhibited and downregulated via cell cycle control at G1 and S phase by epiqafzelechin (Fig. 1), β-sitosterol (Fig. 2) and β-amyrin (Fig. 3), thus can be used as an anticancer therapy for colon tumors. Β-amyrin controls the transcription and proteolytic cleavage of their NH_2_-terminal domain by plasmin and furin proteases and maintains the mRNA stability, thus inhibiting the overexpression of MMPs^58^. The literature reported that HepG2 was first treated with different concentrations of β-amyrin. Then it was exposed to DAPI staining, which depicted that β-amyrin induced apoptosis in Hep-G2 cells, shown by the increased number of cells with white color nuclei at 50 µM β-amyrin concentration^59^. The effect of β-amyrin on the phosphorylation of J.N.K. and p38 was observed, and the results showed increased phosphorylation of both J.N.K. and p38 in a concentration-dependent manner^60^. The levels of antioxidants S.O.D., C.A.T., and G.S.H., which were decreased in the serum due to over-expression of tumor biomarkers, were restored gradually due to targeted therapy of β-sitosterol, β-amyrin, and epiafzelechin. Much research has been conducted on the effect of epiafzelechin-rich extracts on cancer cell lines. Findings demonstrated that epiafzelechin inhibited the proliferation of cancer cell lines in a dose-dependent manner. Epiafzelechin extracted from *acacia tundra* at a concentration of 100 µM revealed the inhibition against the cervical Cancer Hela cells and gastric adenocarcinomas MKN-45 cells^61^. However, 140 mg/g of epiafzelechin demonstrated 20% inhibition of the human bladder cancer TCCUP cell line^62^. Epiafzelechin also depicted necrotic cell death at 130 mg/g concentration in human dermal microvascular endothelial cells. Thus, epiafzelechin at high doses shows antiproliferative activity^63^. However, IL-10 showed a significant indirect relationship with VEGF (r = 0.749*, *p* = 0.009). Rats exposed to NiCl_2_ had disrupted epithelial barrier, as seen by the histopathology of colon sections. In the groups injected phytocompounds containing combinatorial regimen of *β-sitosterol*, *β-amyrin*, and *epiafzelechin*, overall mucosal architecture and graded from the presence of ulcerations, granulation tissue, irregular crypts or the crypt produced by NiCl_2_ were strikingly absent or attenuated (Figs. 10, 11). The histology presentations support the biochemical results, which further demonstrate the therapeutic protection provided by the combinatorial treatment of *β-sitosterol*, *β-amyrin*, and *epiafzelechin* against nickel-induced tissue damage.

To our knowledge, this is the first study to show that adding *β-sitosterol*, *β-amyrin*, and *epiafzelechin* in combination dose as described in the current work to the diet shielded the rat colon from the harmful effects brought on by exposure to NiCl_2_. The data support the medicinal and nutritional potential of the above-discussed phytocompounds with more scientific evidence. Consuming a diet high in *β-sitosterol*, *β-amyrin*, and *epiafzelechin* in suitable doses may help guard against occupational health concerns for persons who work in mining or other related industries where nickel exposure is common.

## Conclusion

The current study concluded through in silico and in vivo analysis that the standardized phytoconstituents β-sitosterol, β-amyrin and epiafzelechin in combination dose regimen offered more potent anti-carcinogenic activity in nickel-induced colon inflammation in rats as compared to the drugs used in chemotherapy with minimum side effects. Therefore, these bioactive compounds in synergism would be more useful as antitumor agents than the individual phytocompounds as well as commercial drugs of chemotherapy and can be used as a better theranostic approach towards treating colon cancer in the future.

## Data Availability

All data generated or analyzed during the study are included in the manuscript.

## References

[1] Shin WS (2016). Programmed activation of cancer cell apoptosis: A tumor-targeted phototherapeutic topoisomerase I inhibitor. Sci. Rep..

[2] Zdrojewicz Z, Popowicz E, Winiarski J (2016). Nickel-role in the human organism and toxic effects. Pol. Merkur. Lek..

[3] Zambelli B, Ciurli S (2013). Nickel and human health. Met. Ions Life Sci..

[4] Afridi HI, Talpur FN, Kazi TG, Brabazon D (2015). Estimation of aluminum, arsenic, lead, and nickel status in the samples of different cigarettes and their effect on human health of Irish smoker hypertensive consumers. Clin. Lab..

[5] Boer JL, Mulrooney SB, Hausinger RP (2014). Nickel-dependent metalloenzymes. Arch. Biochem. Biophys.

[6] Das KK, Reddy RC, Bagoji IB, Das S, Bagali S, Mullur L, Khodnapur JP, Biradar MS (2018). Primary concept of nickel toxicity-an overview. J. Basic Clin. Physiol. Pharm..

[7] Saito M, Arakaki R, Yamada A, Tsunematsu T, Kudo Y, Ishimaru N (2016). Molecular mechanisms of nickel allergy. Int. J. Mol. Sc..

[8] Dukes MP, Rowe RK, Harvey T, Rangel W, Pedigo S (2019). Nickel reduces calcium-dependent dimerization in neural cadherin electronic supplementary information (ESI) available. Metallomics.

[9] Shahzad B, Tanveer M, Rehman A, Cheema SA, Fahad S, Rehman S, Sharma A (2018). Nickel- whether toxic or essential for plants and environment–a review. Plant Physiol. Biochem..

[10] Tawfik O, Sagrillo C, Johnson D, Dey S (1987). Decidualization in the bat: Role of leukotrienes and prostaglandins. Prostaglandins Leukot. Med..

[11] Wolfe, M. M. & Lowe, R. C. Gastric secretions. In *Yamada’s Textbook of Gastroenterology*. Wiley-Blackwell: Hoboken, NJ, USA 399–419 (2015).

[12] Domschke, W., Peskar, B., Holtermüller, K. & Dammann, H. *Prostaglandins and Leukotrienes in Gastrointestinal Diseases*. Springer Science & Business Media: Berlin/Heidelberg, Germany 22–36 (2012).

[13] Osafo, N., Agyare, C., Obiri, D.D. & Antwi, A.O. Mechanism of action of nonsteroidal anti-inflammatory drugs. In *Nonsteroidal Anti-Inflammatory Drugs*. IntechOpen: London, UK 1–15 (2017).

[14] Amadio P, Cummings DM, Amadio P (1993). Nonsteroidal anti-inflammatory drugs: Tailoring therapy to achieve results and avoid toxicity. Postgrad. Med..

[15] Groesch S, Niederberger E, Geisslinger G (2018). Investigational drugs targeting the prostaglandin E2 signaling pathway for treating inflammatory pain. Expert. Opin. Investig. Drugs.

[16] Bell JT (2011). A twin approach to unraveling epigenetics. Trends Genet..

[17] Hou L (2012). Environmental chemical exposures and human epigenetics. Int. J. Epidemiol.

[18] Barouki R (2012). Developmental origins of non-communicable disease: implications for research and public health. Environ. Health.

[19] Xia D (2012). Prostaglandin E2 promotes intestinal tumor growth via D.N.A. methylation. Nat. Med.

[20] Huang SK (2012). Prostaglandin E(2) increases fibroblast gene-specific and global D.N.A. methylation via increased D.N.A. methyltransferase expression. FASEB J..

[21] Peveling-Oberhag J (2012). Dysregulation of global microRNA expression in splenic marginal zone lymphoma and influence of chronic hepatitis C virus infection. Leukemia.

[22] Abdalla MA (2012). Promising candidate urinary microRNA biomarkers for the early detection of hepatocellular carcinoma among high-risk hepatitis C virus Egyptian patients. J. Cancer.

[23] Arzumanyan A (2012). Epigenetic repression of E-cadherin expression by hepatitis B virus x antigen in liver cancer. Oncogene.

[24] Lim JS (2021). Hepatitis C virus core protein overcomes stress-induced premature senescence by down-regulating p16 expression via D.N.A. methylation. Cancer Lett..

[25] Jiang J (2012). Hypomethylated CpG around the transcription start site enables TERT expression, and HPV16 E6 regulates TERT methylation in cervical cancer cells. Gynecol. Oncol..

[26] Hyland PL (2012). Evidence for alteration of EZH2, BMI1, and KDM6A and epigenetic reprogramming in human papillomavirus type 16 E6/E7-expressing keratinocytes. J. Virol..

[27] McLaughlin-Drubin ME (2011). Human papillomavirus E7 oncoprotein induces KDM6A and KDM6B histone demethylase expression and causes epigenetic reprogramming. Proc. Natl. Acad. Sci..

[28] Abdellatif KR (2021). Design, synthesis of new anti-inflammatory agents with a pyrazole core: COX-1/COX-2 inhibition assays, anti-inflammatory, ulcerogenic, histopathological, molecular Modeling, and ADME studies. J. Mol. Struct..

[29] Khodja IA (2020). Design, synthesis, biological evaluation, molecular docking, DFT calculations and in silico ADME analysis of (benz) imidazole-hydrazone derivatives as a promising antioxidant, antifungal, and anti-acetylcholinesterase agents. J. Mol. Struct..

[30] Ivanović V (2020). Lipinski's rule of five, famous extensions and exceptions. Pop. Sci. Artic..

[31] Pagadala NSK, Syed JT (2017). Software for molecular docking: A review. Biophys. Rev..

[32] Cortijo J, Milara J, Mata M, Donet E, Gavara N, Peel SE (2020). Nickel induces intracellular calcium mobilization and pathophysiological responses in human-cultured airway epithelial cells. Chem. Biol. Interact..

[33] Kanwal R, Gupta S (2012). Epigenetic modifications in cancer. Clin. Genet..

[34] Bodily JM (2012). Human papillomavirus E7 enhances hypoxia-inducible factor 1-mediated transcription by inhibiting binding of histone deacetylases. Cancer Res..

[35] Kakkar P, Das B, Viswanathan PN (1984). A modified spectrophotometric assay of superoxide dismutase (SOD). Indian J. Biochem. Biophys..

[36] Moron MS, Depierre JW, Mannervik B (1979). Levels of glutathione, glutathione reductase and glutathione S-transferase activities in rat lung and liver. Biochimica et Biophysica Acta (BBA)-Genl. Sub..

[37] Malik A, Arooj M, Butt TT, Zahid S, Zahid F, Jafar TH, Waquar S, Gan SH, Ahmad S, Mirza MU (2018). In silico and in vivo characterization of cabralealactone, solasodine and Salvadoran in a rat model: Potential anti-inflammatory agents. Drug Design Develop. Ther..

[38] Hsu CH (2012). The HPV E6 oncoprotein targets histone methyltransferases for modulating specific gene transcription. Oncogene.

[39] Sweazea KL, Johnston CS, Knurick J, Bliss CD (2017). Plant-based nutraceutical increases plasma catalase activity in healthy participants: A small double-blind, randomized, placebo-controlled, proof of concept trial. J. Diet Suppl..

[40] Bredt DS, Snyder SH (1994). Transient nitric oxide synthase neurons in the embryonic cerebral cortical plate, sensory ganglia, and olfactory epithelium. Neuron.

[41] Yu H (2012). Epstein-Barr virus downregulates microRNA 203 through the oncoprotein latent membrane protein 1: A contribution to increased tumor incidence in epithelial cells. J. Virol.

[42] Angrisano T (2012). Helicobacter pylori regulates iNOS promoter by histone modifications in human gastric epithelial cells. Med. Microbiol, Immunol..

[43] Sağlık BN, Osmaniye D, Levent S, Çevik UA, Çavuşoğlu BK, Özkay Y, Kaplancıklı ZA (2021). Design, synthesis and biological assessment of new selective COX-2 inhibitors including methyl sulfonyl moiety. Eur. J. Med. Chem..

[44] Alfayomy AM, Abdel-Aziz SA, Marzouk AA, Shaykoon MSA, Narumi A, Konno H, Abou-Seri SM, Ragab FA (2021). Design and synthesis of pyrimidine-5-carbonitrile hybrids as COX-2 inhibitors: Anti-inflammatory activity, ulcerogenic liability, histopathological and docking studies. Bioorg. Chem..

[45] Abdellatif KR, Abdelall EK, Elshemy HA, Lamie PF, Elnahaas E, Amin DM (2021). Design, synthesis of new anti-inflammatory agents with a pyrazole core: COX-1/COX-2 inhibition assays, anti-inflammatory, ulcerogenic, histopathological, molecular modeling, and ADME studies. J. Mol. Struct.

[46] Abolhasani H, Zarghi A, Movahhed TK, Abolhasani A, Daraei B, Dastmalchi S (2021). Design, synthesis and biological evaluation of novel indanone containing spiroisoxazoline derivatives with selective COX-2 inhibition as anticancer agents. Bioorg. Med. Chem.

[47] Bekheit MS, Mohamed HA, Abdel-Wahab BF, Fouad MA (2021). Design and synthesis of new 1, 4, 5-trisubstituted triazole-bearing benzenesulphonamide moiety as selective COX-2 inhibitors. Med. Chem. Res..

[48] Nesaragi AR, Kamble RR, Dixit S, Kodasi B, Hoolageri SR, Bayannavar PK, Dasappa JP, Vootla S, Joshi SD, Kumbar VM (2021). Green synthesis of therapeutically active 1, 3, 4-oxadiazoles as antioxidants, selective COX-2 inhibitors and their in silico studies. Bioorg. Med. Chem. Lett..

[49] Yehiyan A, Barman S, Varia H, Pettit S (2017). Short-course high-dose ibuprofen causing both early and delayed jejunal perforations in a non-smoking man. BMJ Case Rep..

[50] Manali A, Saeidnia S, Ostad SN, Hadjiakhoondi A, Shams Ardekani MR, Vazirian M, Akhtar Y, Khanavi M (2013). Chemical constituents and cytotoxic effect of the main compounds of *Lythrum salicaria* L. Z. Naturforsch.

[51] Shi C, Wu F, Zhu X, Xu J (2013). Incorporation of β-sitosterol into the membrane increases resistance to oxidative stress and lipid peroxidation via estrogen receptor-mediated PI3K/GSK3β signaling. Biochim. Biophysic. Acta.

[52] Radika MK, Viswanathan P, Anuradha CV (2013). Nitric oxide mediates the insulin-sensitizing effects of β-sitosterol in high-fat diet-fed rats. Nitric Oxide.

[53] Saeidnia S, Barari E, Shakeri A, Gohari AR (2013). Isolation and identification of main compounds of Lagochilus cabulicus. Asian J. Chem..

[54] Anyanwu GO, Ur-Rehman N, Onyeneke CE, Rauf K (2015). Medicinal plants of the genus Anthocleista-A review of their ethnobotany, phytochemistry, and pharmacology. J. Etnopharmacol..

[55] Dwivedi A, Sharma K, Sharma YK (2015). Cadamba: A miraculous tree having enormous pharmacological implications. Pharmacogn Rev..

[56] García-Martínez O, De Luna Bertos E, Ramos-Torrecillas J, Manzano-Moreno FJ, Ruiz C (2015). Repercussions of NSAIDS drugs on bone tissue: The osteoblast. Life Sci..

[57] Goldstein JL, Cryer B (2015). Gastrointestinal injury associated with NSAID use: A case study and review of risk factors and preventative strategies. Drug Healthc Patient Saf..

[58] Tovey FI (2015). Role of dietary phospholipids and phytosterols in protection against peptic ulceration as shown by experiments on rats. World J Gastroenterol..

[59] Sheeja MD, Beema SR, Karutha PS, Pandima DK (2017). Cholinesterase inhibitory, anti-amyloidogenic, and neuroprotective effect of the medicinal plant Grewia tiliaefolia–an in vitro and in silico study. Pharm. Biol..

[60] Otto T, Sicinski P (2017). Cell cycle proteins as promising targets in cancer therapy. Nat. Rev. Cancer.

[61] Liu G, Kuang S, Wu S, Jin W, Sun C (2016). A novel polysaccharide from Sargassum integerrimum induces apoptosis in A549 cells and prevents angiogenesis in vitro and in vivo. Sci. Rep.

[62] Wong KC, Cao S, Dong X, Law MC, Chan TH, Wong MS (2017). Epiafzelechin protects against ovariectomy-induced bone loss in adult mice and modulate osteoblastic and osteoclastic functions in vitro. Nutrients.

[63] Abdellatif KR (2021). Design, synthesis of new anti-inflammatory agents with a pyrazole core: COX-1/COX-2 inhibition assays, anti-inflammatory, ulcerogenic, histopathological, molecular Modeling, and ADME studies. J. Mol. Struct..

